# A Bayesian evolutionary model towards understanding wildlife contribution to F4-family *Mycobacterium bovis* transmission in the South-West of France

**DOI:** 10.1186/s13567-022-01044-x

**Published:** 2022-04-02

**Authors:** Hélène Duault, Lorraine Michelet, Maria-Laura Boschiroli, Benoit Durand, Laetitia Canini

**Affiliations:** 1 Epidemiology Unit, Laboratory for Animal Health, Paris-Est University, Anses, 94700 Maisons-Alfort, France; 2 Université Paris-Saclay, Faculté de Médecine, 94270 Le Kremlin-Bicêtre, France; 3 Tuberculosis Reference Laboratory, Bacterial Zoonosis Unit, Laboratory for Animal Health, Paris-Est University, Anses, 94700 Maisons-Alfort, France

**Keywords:** Multi-host system, bovine tuberculosis, genomic epidemiology

## Abstract

In two “départements” in the South-West of France, bovine tuberculosis (bTB) outbreaks due to *Mycobacterium bovis* spoligotype SB0821 have been identified in cattle since 2002 and in wildlife since 2013. Using whole genome sequencing, the aim of our study was to clarify badger contribution to bTB transmission in this area. We used a Bayesian evolutionary model, to infer phylogenetic trees and migration rates between two pathogen populations defined by their host-species. In order to account for sampling bias, sub-population structure was inferred using the marginal approximation of the structured coalescent (Mascot) implemented in BEAST2. We included 167 SB0821 strains (21 isolated from badgers and 146 from cattle) and identified 171 single nucleotide polymorphisms. We selected a HKY model and a strict molecular clock. We estimated a badger-to-cattle transition rate (median: 2.2 transitions/lineage/year) 52 times superior to the cattle-to-badger rate (median: 0.042 transitions/lineage/year). Using the maximum clade credibility tree, we identified that over 75% of the lineages from 1989 to 2000 were present in badgers. In addition, we calculated a median of 64 transition events from badger-to-cattle (IQR: 10–91) and a median of zero transition event from cattle-to-badger (IQR: 0–3). Our model enabled us to infer inter-species transitions but not intra-population transmission as in previous epidemiological studies, where relevant units were farms and badger social groups. Thus, while we could not confirm badgers as possible intermediaries in farm-to-farm transmission, badger-to-cattle transition rate was high and we confirmed long-term presence of *M.* *bovis* in the badger population in the South-West of France.

**Supplementary Information:**

The online version contains supplementary material available at 10.1186/s13567-022-01044-x.

## Introduction

Bovine tuberculosis (bTB) mainly affects cattle, however bTB’s most frequent etiological agent, *Mycobacterium bovis*, can also infect other domestic species as well as wildlife species [1]. *M. bovis* host-species depend on the studied area and the role played by wildlife in these various multi-host systems can sometimes prove to be substantial. Indeed, different wildlife species have been implicated as reservoirs of *M. bovis* around the world; e.g. brush-tailed possums (*Trichosurus vulpecula*) in New Zealand [2] and white-tailed deer (*Odocoileus virginianus*) in Michigan, USA [3]. In Europe, evidence supports badgers (*Meles meles*) in Ireland and Britain [4] and wild boars (*Sus scrofa*) in Spain [5] as bTB reservoirs. In France, wildlife *M. bovis* infection was first detected in red deer (*Cervus elaphus*) and wild boars in Normandy in 2001 [6]. Since then, a national wildlife surveillance program, “Sylvatub” has reported infected badgers and boars in persistent clusters of infection such as the Pyrénées-Atlantiques, Landes and Dordogne “départements” (a French administrative subdivision) as well as infected red deer and roe deer (*Capreolus capreolus*) in Dordogne [7]. In addition, *M. bovis* infection has recently been investigated in red foxes (*Vulpes vulpes*) and infection rates comparable to those in badgers and wild boars were found in Dordogne, Charente and Landes [8].

In the European Union (EU), bTB has been until now subject to control programs (EU directive 64/432/EEC). In France, the program for eradication of bTB in cattle, which started in 1954 was quickly followed by a decrease of bTB herd incidence from 13% in 1965 to <0.1% in 2000 [9]. Following a 6 year period with herd prevalence <0.1%, the officially free of bTB (OTF) status was obtained in 2001. The OTF status mainly presents an economic interest since it facilitates live cattle trade in the EU and with other countries (EU directive 64/432/EEC, [10]). However, this status is currently endangered by persistent clusters of infection, especially in the South-West of France [11]. These past 2 years, the majority of infected herds (68/92 (74%) in 2019 and 84/104 (81%) in 2020) were detected in Nouvelle-Aquitaine (according to the Animal Health Epidemiological platform ESA), a “région”, which contains the Pyrénées-Atlantiques, Landes, Charente and Dordogne, amongst other “départements”.

Systematic *post mortem* inspection of bovine carcasses for lesions compatible with bTB in French abattoirs constitutes the first component of cattle surveillance and periodical herd skin-testing, the second and main component, which currently detects around 70% of bTB clusters (according to the Animal Health Epidemiological platform ESA). Single intradermal tuberculin tests (SITT) or single intradermal comparative tuberculin tests (SICTT) are performed in the cervical region and results are read 72 h post-injection. Herd skin-testing regularity, ranging from annual testing of all animals older than 6 weeks to no testing, is decided at the “département” level. In the Pyrénées-Atlantiques and Landes, herd skin-testing was reinforced in 2012 to an annual regularity in “communes” (i.e. the smallest French administrative subdivision) where bTB outbreaks were detected the previous year. Before the generalization of this annual regularity to all “communes” in 2018, herd skin-testing regularity for the “communes” where bTB outbreaks had not been detected the previous year, was every 2 years in the Landes and every 3 years in the Pyrénées-Atlantiques. Skin-testing is also performed before introduction of all cattle in transit for more than 6 days, coming from at-risk herd, transiting through a high-risk herd with high turnover or coming from a “département” with a 5 year cumulative incidence higher than the national average incidence and when investigating an epidemiological link to a confirmed outbreak.

After culling following a positive skin-testing or after a *post mortem* lesion detection, subsequent PCR testing and bacterial culture are conducted in order to detect mycobacteria. Strains are then sent to the National Reference Laboratory (NRL) where genotypes are determined using spoligotyping and VNTR (Variable Number Tandem Repeat) typing methods, this has led to the identification of regional genotypes [12]. Positive identification of a bTB case in a farm leads to an official declaration of infection and control measures are then implemented; depending on the control strategy, this official declaration of infection could cause long-term depopulation of vulnerable cattle farms [13].

Following the report of *Mycobacterium bovis* infection in red deer and wild boars in 2001, with genotyping linking this wildlife outbreak to cases in nearby cattle [6] and the discovery of infected wildlife in other affected regions elsewhere in France, “Sylvatub”, was started in 2011 to investigate bTB infection in badgers, boars, red deer and roe deer [7]. “Sylvatub” submits road-kill, hunting carcasses and animals captured in annual campaigns designed at the “département” level, to a protocol similar to cattle surveillance, i.e. PCR testing, bacterial culture and genotyping [7].

The majority of *M. bovis* detection in wildlife are located in the vicinity of cattle outbreaks and present the same genotypes [14]. A current example of this can be found in the Pyrénées-Atlantiques and Landes “départements”, where “Sylvatub” was started in 2012 and surveillance data reported two spoligotypes belonging to the F4-family/cluster A [15, 16] shared by cattle, badgers and wild boars: SB0821 and SB0832 [14]. In the Pyrénées-Atlantiques and Landes, the number of newly infected herds declared each year ranged from 16 to 31 between 2012 and 2017, without any obvious trend (Boschiroli, personal communication). Moreover, the apparent prevalence of bTB in badgers in the region was estimated at 5.9% [3.9–6.8%] 95% CI in 2013–2014 (by culture) and 7.9% [5.2–11.2%] 95% CI in 2016–2017 (by PCR testing) [7].

Since the same genotype profile is shared by both cattle and wildlife, a more discriminating method to differentiate strains is necessary in order to understand transmission dynamics. Whole genome sequencing data has been previously selected for its higher resolution to investigate bTB transmission [17, 18].

When studying transmission dynamics between different populations, a Bayesian evolutionary model applied to *M. bovis* transmission between cattle and wildlife, while not always conclusive on the direction of transmission [17], has recently brought insights to badger intervention in bTB transmission in the UK [19]. In a Bayesian evolutionary model, genetic sequences are annotated by a state e.g. geographical locations [20, 21] or host-species [22, 23]. Reconstruction of ancestral node states in the phylogeny enables estimation of migration processes between populations. In this study, our aim was to analyze whole genome sequencing data using a Bayesian evolutionary model in order to better understand badger contribution to transmission in a SB0821 bTB multi-host system, in the Pyrénées-Atlantiques and Landes French “départements”.

## Materials and methods

### Study area and data collection

**Figure 1:**
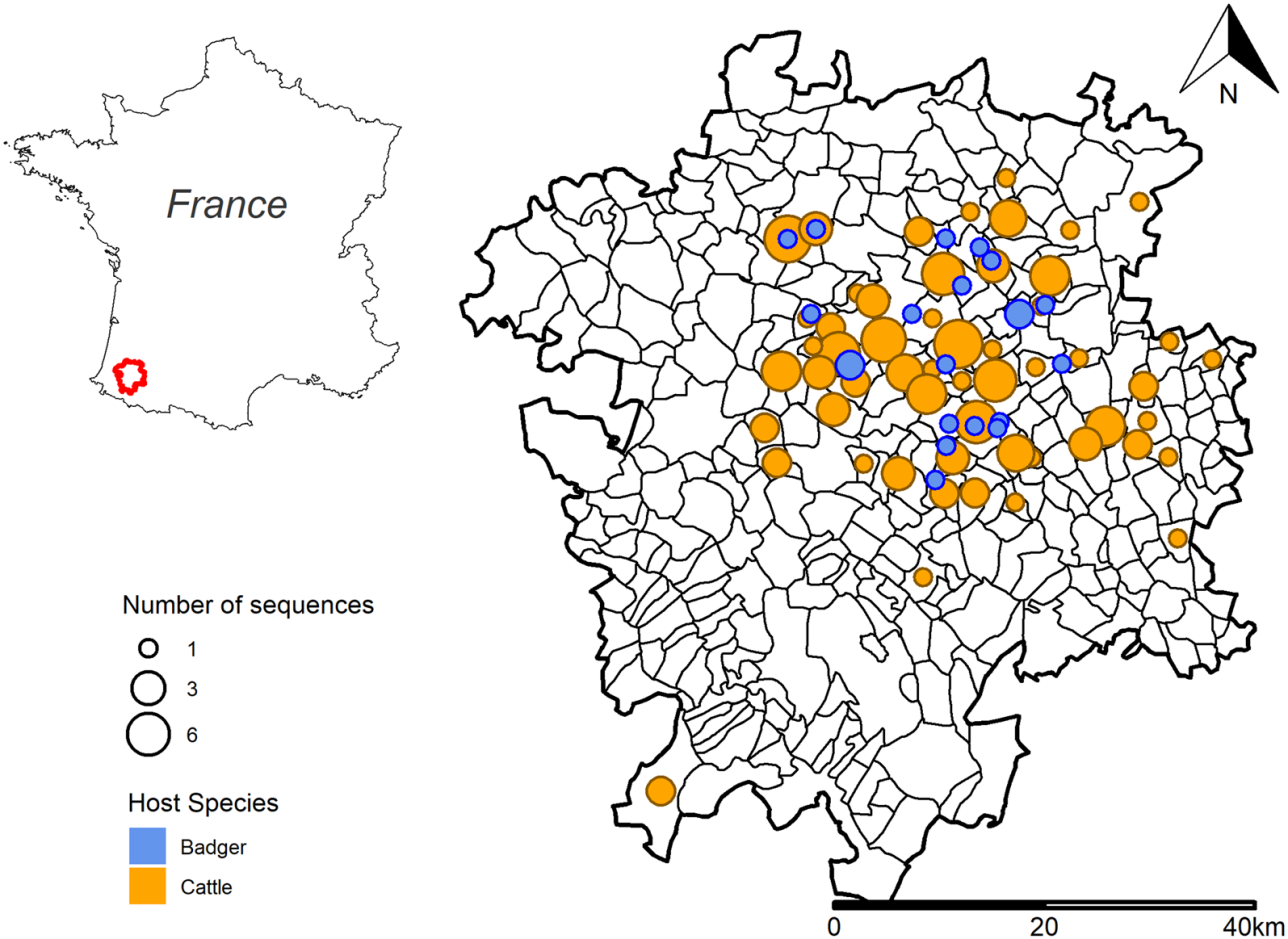
**Study area with the location of isolated SB0821 strains according to host-species and number of cases.** Black polygons represent “commune” limits. Colors represent host-species.

Our study area consisted of the “communes” selected in previous works on the badger-cattle bTB system in the South-West of France [24, 25]. This study area was restricted to a 3754 km^2^ area of 335 “communes” (Figure 1), straddling the border of Pyrénées-Atlantiques and Landes.

A maximum of three SB0821 strains per official declaration of infection per farm, collected between 2002 and 2017, were included in the study. All SB0821 badger strains collected during our study period by “Sylvatub” were included.

The sampling date considered was either the date of slaughter for cattle strains or the date of capture recorded by “Sylvatub” for badger strains. Cattle information was provided by the “Base de données nationale d’identification*”* (BDNI), in which every bovine is registered in France. BDNI records date of birth, date of death, cattle movements and their cause (e.g*.* trade, slaughter).

### Genomic data

Upon reception at the NRL, liquid culture media (7H9 + ADC) was employed to grow the *M. bovis* strains. After a heating step, the lysate obtained was sent for purification and Illumina sequencing (paired-end 2*150 bp) to the Paris Brain Institute (ICM). At the ICM, sequencing quality was controlled using FASTQC with an acceptability Phred score threshold of 30. Sequence alignment and Single Nucleotide Polymorphism (SNP) calling were computed at the NRL using the AF2122/97 reference strain on Bionumerics software, version 7.6 (AppliedMath, Belgium). SNPs identified were selected according to strict criteria of wgSNP module: (i) they had to be present on at least 5 reads in both forward and reverse direction, (ii) 12 base pairs had to separate them, (iii) they were not present in repetitive regions of the genome and (iv) ambiguous SNPs (at least one unreliable (N) base, ambiguous (non ATCG) base or gap) were not included. SNPs were then used to reconstruct a maximum parsimony tree on Bionumerics in order to identify genetic outliers. We estimated pairwise distances between sequences using the *dist.dna* function available in the *ape* package version 5.0 [26] on R4.0.3. The model considered was the F84 model [27] since it closely resembles the HKY model [28] that was previously used on *M. bovis* strains [17, 29]. Distances were estimated between all, badger and then cattle sequences.

### Bayesian evolutionary model

We used a Bayesian evolutionary model consisting of a structured coalescent population model, in order to capture the transition between two pathogen populations defined by their host-species, using BEAST2 (Bayesian Evolutionary Analysis by Sampling Trees) v2.6.3 [30]. The probability of nucleotide substitutions was described by the substitution model and the molecular clock modeled the evolution of substitution rates across branches. Moreover, the pathogen sub-population structure was inferred using the marginal approximation implemented in the Mascot package v2.1.2 [21].

We needed to select the most appropriate substitution and molecular clock models. Models were finally compared using the Bayes Factor (BF) after estimating their marginal likelihoods with the “Nested Sampling” algorithm implemented in the NS package v1.1.0 [31]. In the NS estimation, the number of particles was *N* = 1 (or 10 if results were inconclusive with *N* = 1) and subchain length was fixed to 100 000. We first tested three substitution models: the Jukes-Cantor (JC) model [32], in which all substitutions are equally likely and base frequencies are equal, the Hasegawa-Kishino-Yano (HKY) [28], in which substitution probabilities depend on the nature of bases and all base frequencies differ, and Generalized-Time-Reversible (GTR) [33] model, where all substitution probabilities and base frequencies are independent. We then tested three molecular clock models: the strict clock with constant substitution rates across branches [34], and the relaxed uncorrelated lognormal and exponential clocks with substitution rates varying over branches [35]. All models were tested in BEAST2 software after annotation on BEAUti interface [30]. We set the site model frequencies parameter to empirical and the constant effective population prior to a lognormal distribution (mean: 0, standard deviation: 1); other parameters kept their default settings. We selected a chain length of 300 million iterations, a burn-in period of 10% and a sampling frequency of 1 in 30 000. Four replicates were performed and combined in LogCombiner v.2.6.3 with a lower sampling frequency of 1 in 120 000.

We checked for convergence i.e. stationary distribution of the MCMC (Markov chain Monte Carlo), and independence of sampling (Effective Sample Size (ESS) above 200 for each parameter), on TRACER v1.7.1 [36]. To summarize the posterior sampled trees, a maximum clade credibility (MCC) tree was built via Tree Annotator using the common ancestor heights option [37]. In the MCC tree, host-species of internal nodes were considered unknown if the posterior probability of their “host” (i.e. “Badger” or “Cattle”) was lower than 0.70, otherwise three host-species probability categories were represented: ]0.7; 0.8], ]0.8; 0.9] and ]0.9; 1]. We then inferred the lineages’ host-species through time by considering that state transition between two nodes occurred at the parental node. The MCC tree was visualized on R4.0.3 with treeio [38] and ggtree packages [39].

In addition, we resampled the posterior trees at a frequency of 1 in 1 200 000 using LogCombiner and imported the resulting 1004 trees in R. In a phylogenetic tree, we have information on the host-species (badger or cattle) of each node. We can therefore count the number of times a parental node and a descendant node do not belong to the same host-species. This number corresponds to inter-species lineage transitions (badger-to-cattle and cattle-to-badger). However, when two consecutive nodes belong to the same host-species, we cannot infer with our method whether the lineage remains in the same animal, in the same group of animals (social group for badgers, farm for cattle), or if there is one or multiple within-species transmission events, within and/or between groups of animals. Similarly, between two nodes hosted by different species, at least one transmission event took place (between a badger and cattle) however, other transmission events could have taken place. Therefore for each tree, we counted the number of inter-species lineage transitions, the number of times lineages remained in the same host-species between two nodes (which we called intra-species persistence) as well as the number of unknown transitions, i.e. the number of times one or both consecutive nodes are considered unknown (host-species probability lower than 0.70). We then calculated the proportion of lineage transitions through time by summing the number of each transition type per year divided by the number of transitions occurring in that year. We considered that state transition between two nodes occurred at the parental node and dated these transitions using the *castor* package version 1.7.0 [40]. Thus, the number of transitions through time corresponds to the sum of internal nodes dated from each year multiplied by two (since one node diverges into two lineages). So transition from an internal node to a tip (representing the isolates) will not be represented at the time of sampling of isolates but at the time of the internal node, that immediately precedes the isolate in the tree. Transition from an internal node to a tip representing a cattle (badger) isolate could correspond to either a badger-to-cattle (cattle-to-badger) transition, an unknown transition or cattle (badger) persistence.

Similarly to the MCC tree, three probability thresholds were used to determine the host-species of internal nodes: 0.7, 0.8 and 0.9. However, since all 1004 resampled trees are studied rather than the consensus tree, the most recent common ancestor (MRCA) of some trees can be dated from before the MRCA of the MCC tree. Therefore, the time range considered is wider than for the MCC tree.

## Results

### Genomic data

**Figure 2:**
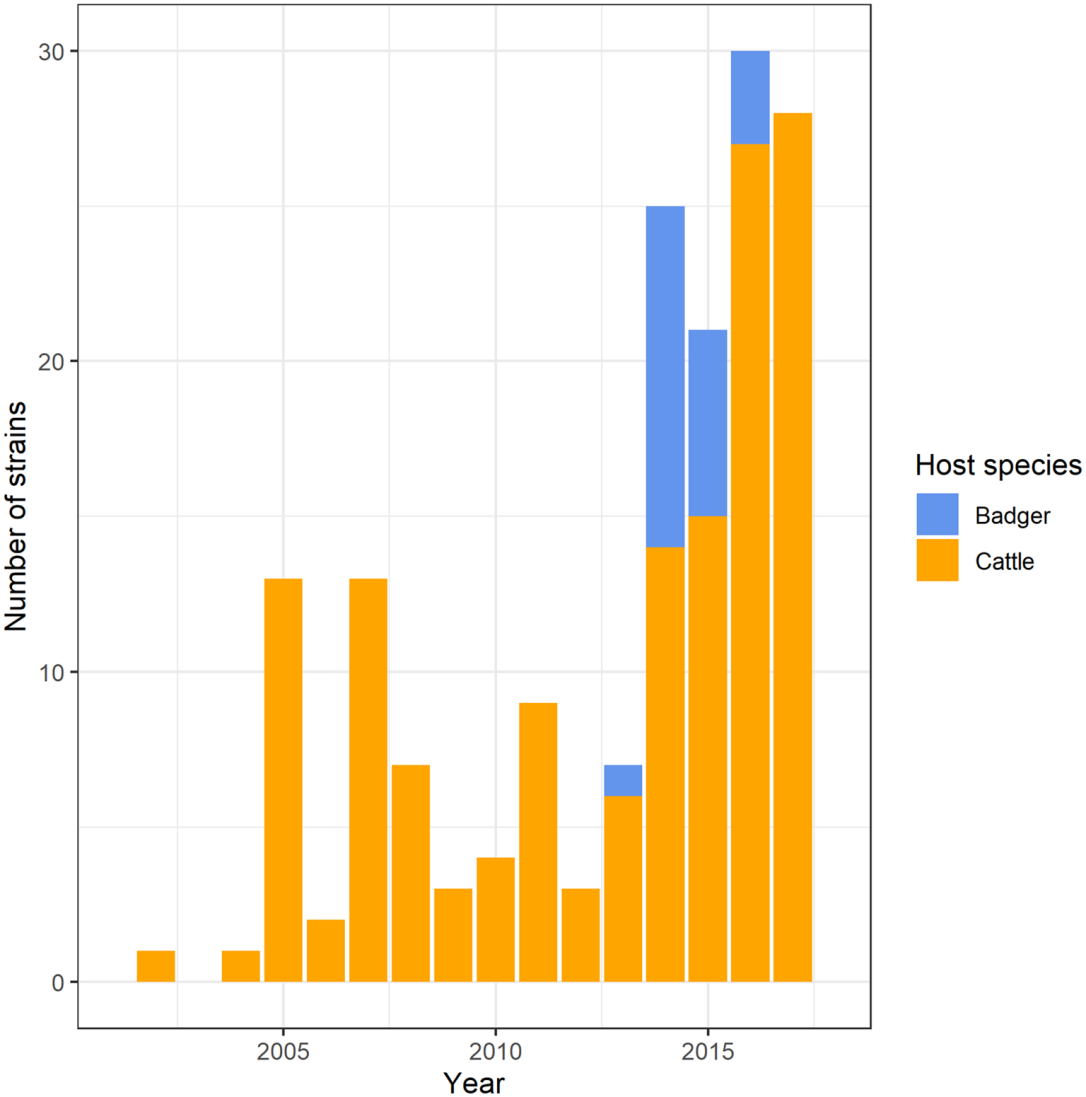
**Number of SB0821 strains isolated by year of death.** Colors represent host-species.

From 167 SB0821 strains, 171 SNPs were identified. Pairwise distances between sequences ranged from 0 to 0.145 for all and cattle strains (median: 0.042) and from 0 to 0.108 for badger strains (median: 0.036). Among these 167 SB0821 strains, 146 were isolated from cattle and 21 from badgers (Figure 2). In 2002, SB0821 strains were first detected in cattle, which preceded our first SB0821 badger strain (2013).

### Bayesian evolutionary model

We selected a strict molecular clock based on the BF comparisons (Additional file 1). However, we could not differentiate between the HKY and GTR substitution models; HKY was chosen based on previous works [17, 29].

The median transition/transversion ratio (kappa) parameter of the HKY model was estimated at 5.9 (95% HPD: “High Posterior Density”: [4.2; 8.2]) (Additional file 2). Estimations of median substitution rate and tree height were respectively 0.41 substitutions/genome/year (95% HPD: [0.29; 0.55]) and 27.5 years (95% HPD: [21.0; 36.6]). Therefore, the MRCA was estimated to have been circulating in 1990 (95% HPD: [1980; 1996]). The model estimated a badger-to-cattle transition rate (median of 2.2 transitions/lineage/year, 95% HPD: [0.74; 4.5]) 52 times superior to the cattle-to-badger transition rate (median 0.042 transitions/lineage/year, 95% HPD: [3.5 × 10^–5^; 0.24]). Estimation of effective population sizes Ne was higher for the badger population (median of 34, 95% HPD: [20; 51]) than for the cattle population (median of 1.2, 95% HPD: [0.27; 2.7]) (Additional file 2).

**Figure 3:**
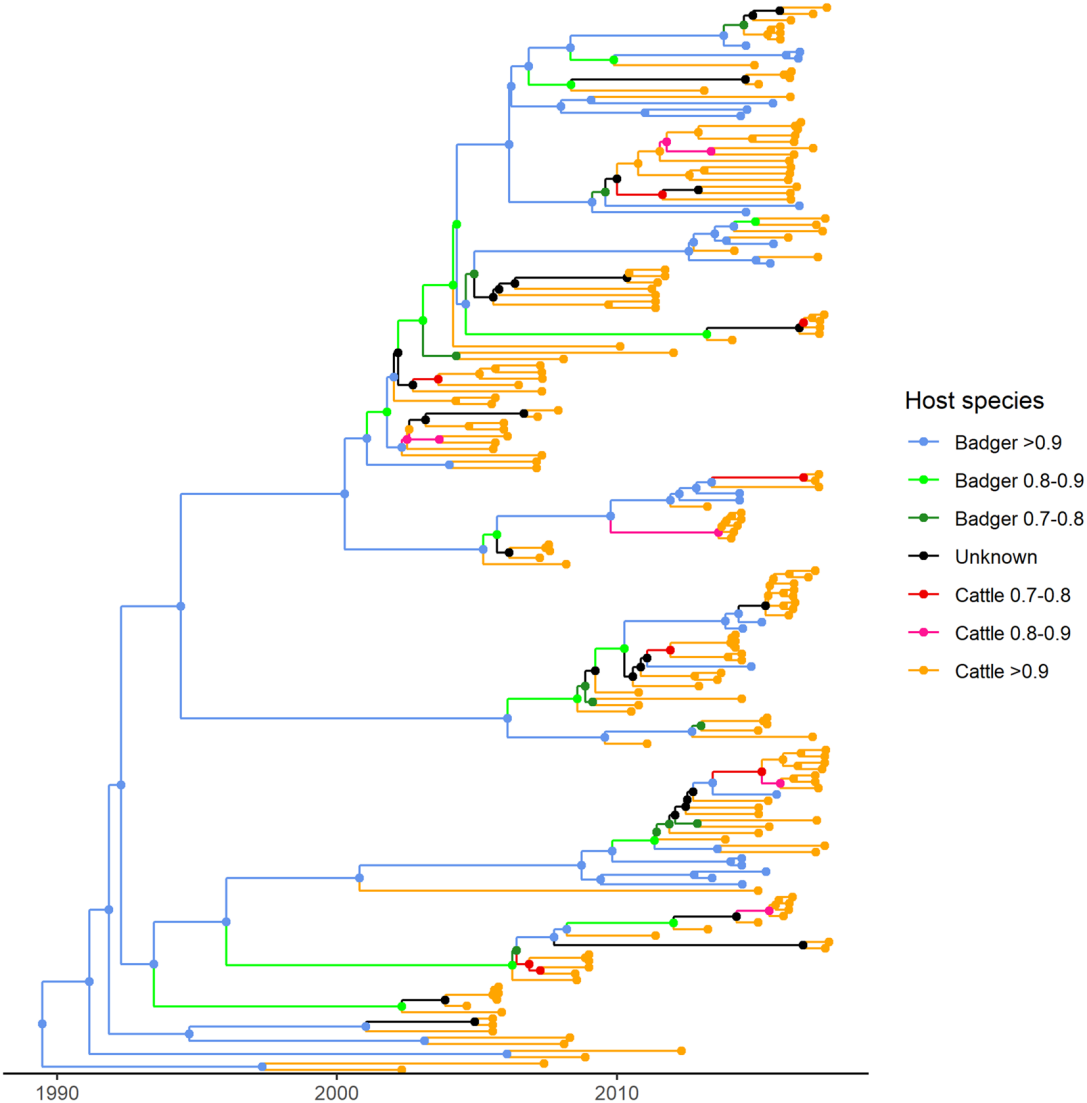
**Maximum Clade Credibility (MCC) tree reconstructed with 146 SB0821 strains isolated in cattle and 21 isolated from badgers.** Colors represent either host-species, in which the strains were isolated (for tree tips) or the reconstructed host-species (internal nodes). Host-species are considered unknown if the host probability is inferior to 0.70.

**Figure 4:**
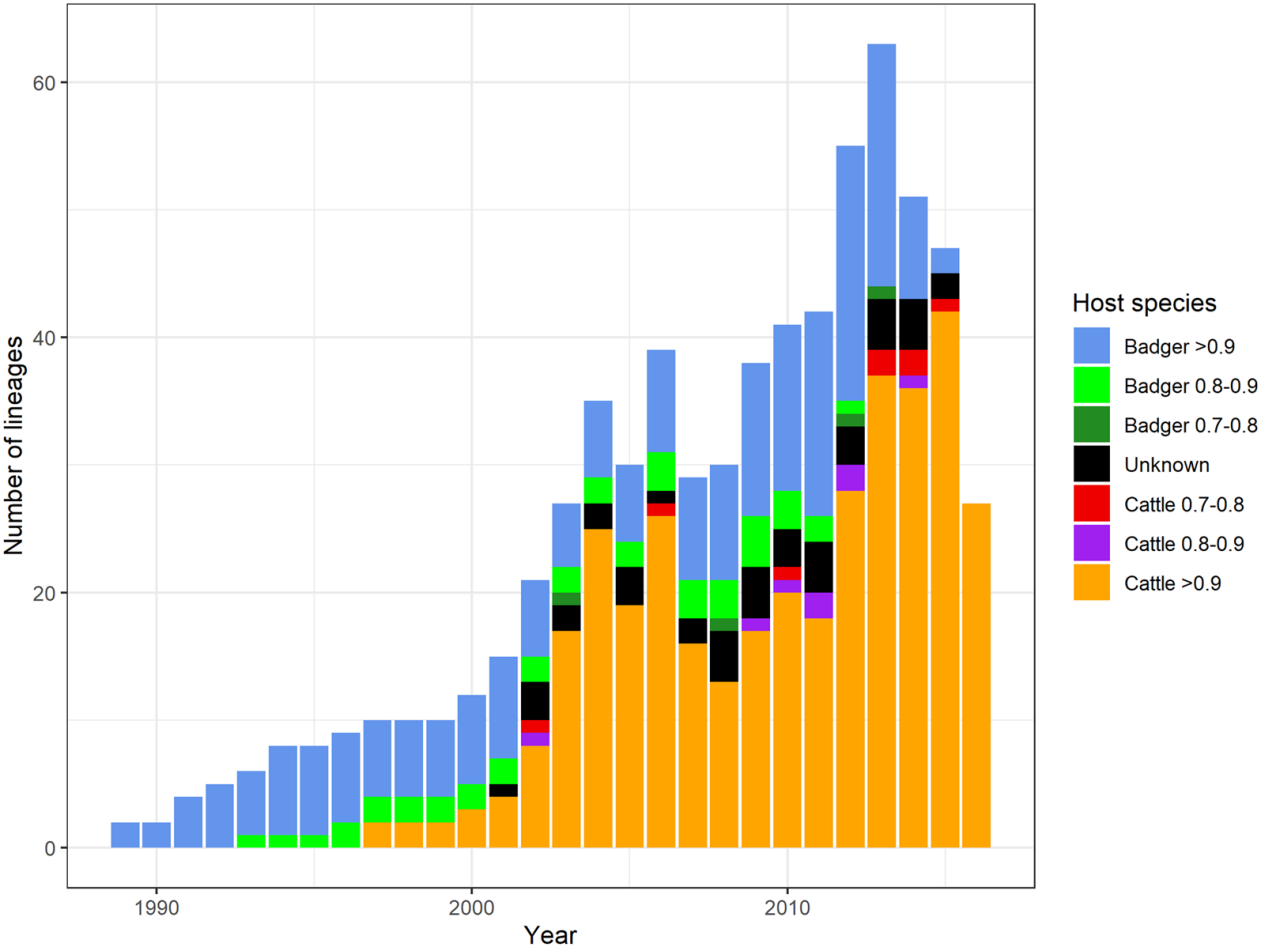
**Host-species of lineages through time estimated in the Maximum Clade Credibility (MCC) tree.** Colors represent host-species and posterior probability. Host-species are considered unknown if the host probability is inferior to 0.70.

In the MCC tree (Figure 3 and Additional files 3, 4), 81 out of 166 internal nodes including the root (with a host probability equal to 0.94) and the nodes closest to the root were identified as hosted by badgers (55 with a posterior probability > 0.9, 15 with a posterior probability between 0.8 and 0.9 and 11 between 0.7 and 0.8). Among the remaining 85 internal nodes, host-species were identified as cattle for 57 nodes (42 with a posterior probability > 0.9, 7 with a posterior probability between 0.8 and 0.9 and 8 between 0.7 and 0.8) and unknown for 28. Figure 4 depicts the host-species of lineages in the MCC tree through time, lineages were estimated to have been circulating solely in badgers until 1996 and over 75% of lineages per year were present in badgers until 2000. Moreover, we predicted two peaks of cattle lineages, one occurring in the mid-2000s and another in the mid-2010s, following a badger peak.

Among the 1004 resampled trees, the median tree height was estimated and corresponded to a MRCA circulating in 1990 (1^st^ quartile: 1978, 3^rd^ quartile: 1996). Moreover, in these trees and when considering the 0.9 probability threshold, we calculated a median of 64 badger-to-cattle transition events (1^st^ quartile: 10, 3^rd^ quartile: 91) and zero cattle-to-badger transition (1^st^ quartile: 0, 3^rd^ quartile: 3). However, the number of times a lineage persists in the same host-species are similar when considering the badger (median: 109, 1^st^ quartile: 14, 3^rd^ quartile: 137) and the cattle population (median: 112, 1^st^ quartile: 78, 3^rd^ quartile: 158). This asymmetry between the number of inter-species transitions as well as the similarity between the intra-species persistence were observed for the three different thresholds (Additional file 5).

**Figure 5:**
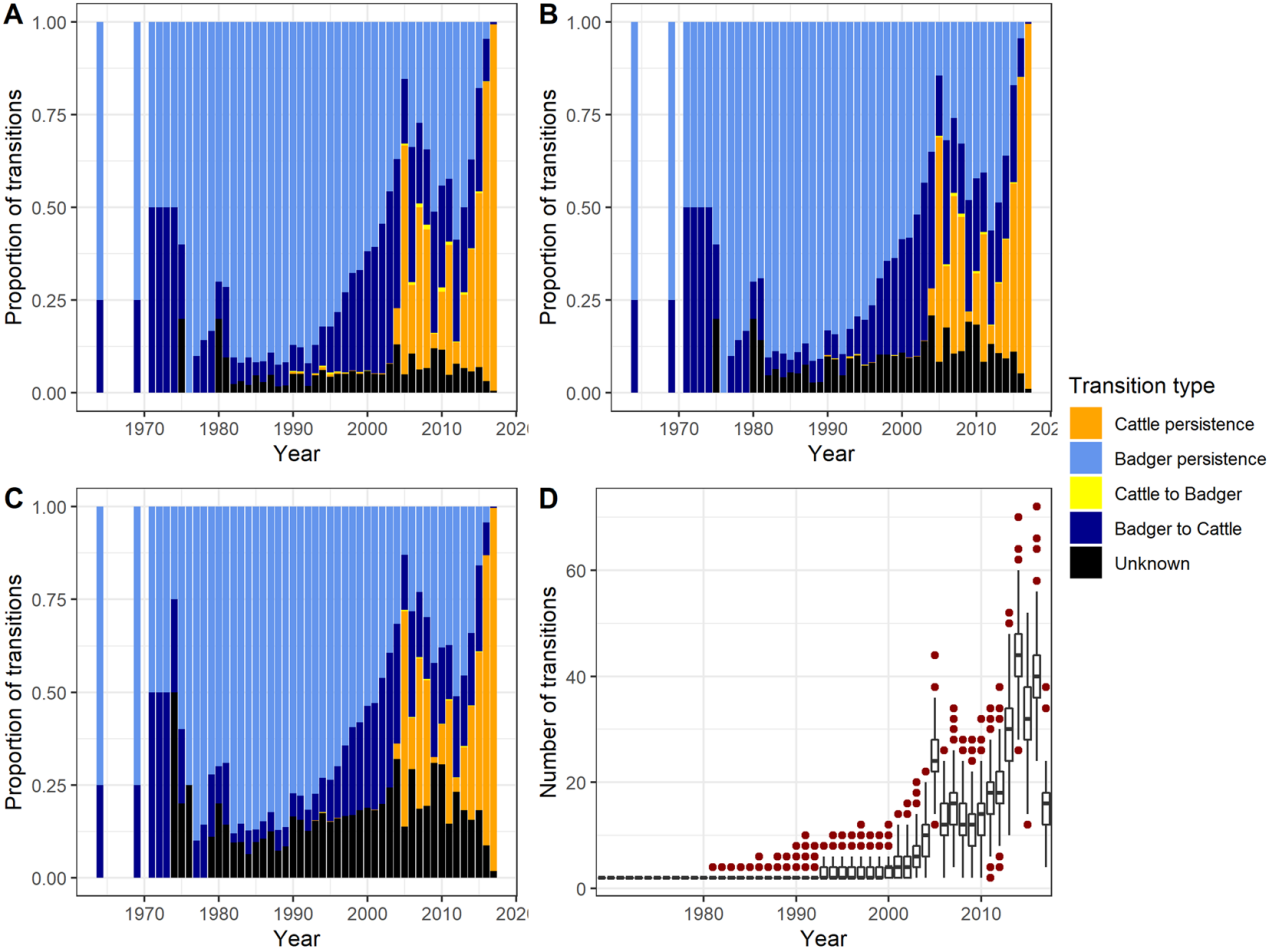
**Proportion of lineage transitions estimated in the 1004 resampled trees.** We used a 0.7 (**A**), 0.8 (**B**) and a 0.9 (**C**) probability threshold. In (**D**), the number of transitions per tree from the 1004 trees is represented over time. Colors represent transition type.

Figure 5 shows that the type of lineage transitions observed in the 1004 trees varies over time. The proportion of badger persistence constituted over 50% of lineage transitions from 1964 to 2001 (excepted in 1974, where 50% of lineages were unknown with the 0.9 probability threshold) while cattle persistence started in 1990 at the earliest. Cattle persistence represented over 50% of transitions in 2005 and again in 2016 and 2017, which corresponds to the dates of the two cattle lineage peaks in the MCC tree. In addition, the proportion of cattle-to-badger transitions never exceeded 1.3% of lineage transitions.

## Discussion

In this work, we used whole genome sequencing data in order to investigate the role played by badgers in SB0821 *M. bovis* strains transmission in the Pyrénées-Atlantiques and Landes. This region situated in the South-West of France is of major interest concerning bTB control in the country since it has been continuously harboring persistent clusters of infection, especially in the past decade (according to surveillance data available on the Animal Health Epidemiological platform ESA).

For the Bayesian evolutionary model, we selected a strict molecular clock. We also chose a HKY substitution model over GTR according to past Bayesian studies [17, 29]. Estimated substitution rate of 0.41 substitutions/genome/year (95% HPD: [0.29; 0.55]) was higher than estimations from past studies in Northern Ireland (0.15, 95% HPD: [0.04–0.26] [41] and 0.2, 95% HPD: [0.1–0.3] [42]) and in Michigan, USA (0.2, 95% HPD: [0.1–0.3] [29]). However, Crispell et al. in 2017 [17] estimated a higher rate of 0.53 substitutions/genome/year, 95% HPD: [0.22–0.94]. Observed differences between these studies could be attributed to the *M. bovis* lineage; specific lineage characteristics have been highlighted in *M. tuberculosis* [43]. *M. bovis* strains studied by Biek et al. and Trewby et al. [41, 42] are part of the Eu1 clonal complex [44]. In France, *M. bovis* lineages differ according to the area studied [15], our estimations were based on SB0821 strains, which belong to the F4-family/cluster A [16]. Lastly, studies based on *M. bovis* strains, which did not share the same spoligotype nor VNTR profile [17] could estimate a higher substitution rate. Variation between estimations could also depend on the sampled host-species. Wildlife species studied vary, e.g*.* the white-tailed deer and the elk in Michigan [29] or even the badger in Northern Ireland [41, 42].

Bayesian inference methods are used to reconstruct ancestral node states (e.g. in our case, host-species) and to estimate parameters such as inter-species transition rates. In order to mitigate the bias due to sampling process in the migration rate estimation, we chose to use a structured coalescent method. More specifically, we used the Marginal Approximation of the Structured COalescenT (MASCOT) to model the evolution of SB0821 strains isolated from cattle and badgers [21]. This method assumes a constant effective population size over time. The past demographics until 2017 are difficult to estimate in the region due to the late implementation of the wildlife surveillance system in 2012. The discovery of bTB in wildlife caused an increase in cattle surveillance and thus an increase in bTB detection in cattle. However, the apparent prevalence in badgers did not seem to vary significantly between the two estimations (2013–2014 and 2016–2017) by Réveillaud et al. [7] and the number of newly infected farms did not follow an obvious trend over the same period of time (Boschiroli, personal communication).

Similar genetic distances were estimated between cattle and badger sequences. However, considering the fact that this was based on 21 badger sequences and 146 cattle sequences, this means that badger strains presented a higher genetic diversity. This was consistent with the higher effective population size estimated in the badger population compared to the cattle population.

The average badger density in 13 study sites (including a 50 km^2^ site situated in our study area) in France was estimated at 3.8 badgers per km^2^ (range: 1.7–7.9), which is “relatively lower than those found in the UK and concordant with global estimates from Ireland” [45]. We inferred a badger-to-cattle transition rate 52 times greater than the cattle-to-badger rate. Our results are consistent with a previous study by Crispell et al. showing a badger-to-cattle transition rate (0.045 transitions/lineage/year) 10 times superior to the cattle-to-badger transition rate (0.0044 per lineage per year) on a subset of cattle (*n* = 83) and badger (*n* = 97) strains isolated in the UK [19]. Conversely, Rossi et al. estimated a higher cattle-to-badger transition rate in a newly infected region in the North-West of England and concluded on the possible requirement of a “build-up in badger infections […] before badger-to-cattle infections become probable” [46].

Crispell et al. estimated similar results than in our work concerning inter-species transmission events comprised mainly of badger-to-cattle transmission and a median of zero cattle-to-badger transmission. Moreover, the variation of lineages’ host-species through time in our consensus tree showed an increase in cattle lineages following an increase in badger lineages. These results suggest that badger-to-cattle transmission may be amplified by onward cattle-to-cattle transmission, a hypothesis proposed by Donnelly and Nouvellet in the UK [47]. Similarly, a study that analyzed an empirical contact network of cattle farms in the same region, concluded on the importance of badger-mediated contacts in bTB spread [24].

Crispell et al. had an interesting approach and estimated the minimum number of intra-species and inter-species transmission events [19]. To this end, the authors assumed that a coalescent event corresponded to at least one transmission event. Therefore, the existence of a single pathogen lineage within an infected animal was implied. We did not make any assumptions on the within-host evolution nor on the timing of transmission. While we considered an inter-species transition to correspond to at least one transmission event between cattle and badger, we did not estimate the number of within-species transmission events. However, the majority of transitions being identified as persistence suggests the importance of intra-species transmission events highlighted in Crispell et al.’s work [19]. Indeed, the majority of the lineage transitions until 2001 were identified as badger persistence, which either correspond to the evolution of *M. bovis* lineages in the same badger or bTB transmission between badgers belonging to the same social group (i.e*.* sett) or to neighboring setts. Conversely, Bouchez-Zacria et al. used a stochastic model of *M. bovis* transmission within the badger-cattle system in the Pyrénées-Atlantiques and Landes and determined limited inter-social group bTB transmission [48]. Therefore, the majority of badger-to-badger persistence until 2001 suggest either long-term carriage of bTB in a badger and/or a long transmission history within a social group.

Similarly to badger persistence, cattle persistence could either correspond to the evolution of *M. bovis* lineages in the same animal, intra-farm or between-farm bTB transmission. Trade data provided by the BDNI determined the significant role of cattle movements in bTB transmission in the Pyrénées-Atlantiques and Landes [24], which could explain between-farm bTB transmission. However, transmission modeling of this badger-cattle system determined that 49.3% of farm infections were due to proximity to pastures belonging to an infected farm [48].

Nonetheless, badger-to-cattle and cattle-to-cattle transmissions are not the only possible source of farm infection. In these two models [24, 48] as well as in our work, contribution of other wildlife species were not included. Infected wild boars have been detected in the Pyrénées-Atlantiques and Landes since the implementation of “Sylvatub” in the area. While a badger movement study in Europe estimated a mean distance of 1.7 km traveled by badgers with some rare long distance travels of up to 22 km [49], the mean daily distances traveled by wild boars is estimated to be around 7–13 km [50]. Therefore, wild boars could have contributed to *M. bovis* spread in a way badgers, typically traveling shorter distances, could not have.

We used genomic data to study the role of badgers in bTB transmission in the Pyrénées-Atlantiques and Landes. However, the sampling process differs between cattle and wildlife strains. According to expert opinions, possible environmental contamination and deterioration of wildlife carcasses could lower the culture sensitivity by 35% and since wildlife samples are pooled, PCR test sensitivity could decrease by 15% [51].

Moreover, in practice, while herd skin-testing rhythm varies between “communes”, cattle surveillance concerns all animals over 24 months. In our study area, while the testing of road-killed badgers did not depend on the presence of bTB in cattle, badger capture protocol changed over the years and varied from one place to another according to the detection of nearby cases in cattle. Contrary to the registered and easily accessible cattle population, the entirety of the badger population cannot be surveilled for practical (free-ranging population) and financial reasons, which contributes to an unavoidable underestimation of cases. However, our choice in the MASCOT method was motivated by the fact that it does not treat the number of each host-species as data and thus helps reduce the impact of sampling bias [52].

In conclusion, our Bayesian evolutionary model enabled us to infer inter-species (badger-to-cattle and cattle-to-badger) transitions but not intra-species transmission as in previous epidemiological studies, where relevant units were farms and badger social groups. Therefore, we could not confirm badger social groups as possible intermediaries in farm-to-farm transmission. However, our results highlighted long-term *M. bovis* presence in the badger population and a high badger-to-cattle transition rate in the Pyrénées-Atlantiques and Landes, which justifies control measures implemented to prevent contacts between cattle and badgers. Further research including transmission tree reconstruction of this multi-host system could help us better understand intra-species bTB transmission and integrate the contribution of other wildlife species in this bTB multi-host system. Including further genomic data isolated from cattle, badgers and especially wild boars would improve our work.

## Supplementary Information

**Additional file 1:** **Model selection with the Bayes Factor.** N corresponds to the number of particles, ML1 (2) to the log maximum likelihood of model 1 (2) and SD to the standard deviation. Log(BF) is the difference between ML1 and ML2. If (BF) > 0 (< 0) than model 1 (2) is favored. “-” means that the results were inconclusive. Subpopulations defined by host-species are not taken into account.

**Additional file 2:** **Parameters estimation (median and 95% High Posterior Density (HPD) interval) and effective sample size (ESS).** Ne is the effective population size and ESS stands for effective sample size.

**Additional file 3:** **Maximum Clade Credibility (MCC) tree reconstructed with strain names.** Colors represent either host-species, in which the strains were isolated (for tree tips) or the reconstructed host-species (internal nodes). Host-species are considered unknown if the host probability is inferior to 0.70.

**Additional file 4:** **Maximum Clade Credibility (MCC) tree reconstructed with posterior probabilities.** Colors represent the posterior probability of the nodes.

**Additional file 5:** **Number of inter-species transitions per tree calculated over 1004 sampled trees.** Number of transitions are represented according to transition type and various probability thresholds (0.7, 0.8 and 0.9).

**Additional file 6:** **List of sample names, host-species and their accession numbers.**

## Data Availability

All WGS data used for these analyses have been uploaded to the European Nucleotide Archive (PRJE45853). The individual isolates can be accessed under the following Biosample accession numbers: SAMEA8939070-SAMEA8939236 (see Additional file 6).
