# Supplementary material for: A Bayesian evolutionary model towards understanding wildlife contribution to F4-family Mycobacterium bovis transmission in the South-West of France
Source: Vet Res. 2022 Apr 2;53:28. doi: 10.1186/s13567-022-01044-x (PMC8976416; doi:10.1186/s13567-022-01044-x)
Supplement: Supplementary file 1 — Additional file 1: Model selection with the Bayes Factor. N corresponds to the number of particles, ML1 (2) to the log maximum likelihood of model 1 (2) and SD to the standard deviation. Log(BF) is the difference between ML1 and ML2. If (BF) > 0 (< 0) than model 1 (2) is favored. “-” means that the results were inconclusive. Subpopulations defined by host-species are not taken into account. [file 13567_2022_1044_MOESM1_ESM.docx]

| Model 1 | Model 2 | N | ML1 | ML2 | SD1 | SD2 | Log(BF) | Model favored (chosen) |
| --- | --- | --- | --- | --- | --- | --- | --- | --- |
| JC substitution model  Strict molecular clock  Constant population | HKY substitution model  Strict molecular clock  Constant population | 10 | -1995,24 | -1933,18 | 6,84 | 6,96 | -62,06 | **Model 2** |
| HKY substitution model  Strict molecular clock  Constant population | GTR substitution model  Strict molecular clock  Constant population | 10 | - | - | - | - | - | **(Model 2)** |
| HKY substitution model  Strict molecular clock  Constant population | HKY substitution model  Exponential relaxed clock  Constant population | 1 | -1894,05 | -39504,85 | 21,57 | 6,29 | 37610,8 | **Model 1** |
| HKY substitution model  Strict molecular clock  Constant population | HKY substitution model  Log normal relaxed clock  Constant population | 1 | -1894,05 | -2203,36 | 21,57 | 22,97 | 309,31 | **Model 1** |
| HKY substitution model  Strict molecular clock  Constant population | HKY substitution model  Strict molecular clock  Exponential population | 10 | -1933,18 | -2004,58 | 6,96 | 7,4 | 71,4 | **Model 1** |
| HKY substitution model  Strict molecular clock  Constant population | HKY substitution model  Strict molecular clock  “Skyline” population | 10 | -1933,18 | -1958,8 | 6,96 | 7,03 | 25,62 | **Model 1** |
