# Supplementary material for: A Bayesian evolutionary model towards understanding wildlife contribution to F4-family Mycobacterium bovis transmission in the South-West of France
Source: Vet Res. 2022 Apr 2;53:28. doi: 10.1186/s13567-022-01044-x (PMC8976416; doi:10.1186/s13567-022-01044-x)
Supplement: Supplementary file 2 — Additional file 2: Parameters estimation (median and 95% High Posterior Density (HPD) interval) and effective sample size (ESS). Ne is the effective population size and ESS stands for effective sample size. [file 13567_2022_1044_MOESM2_ESM.docx]

| Parameters | Median | Lower bound 95% HPD | Upper bound 95% HPD | ESS |
| --- | --- | --- | --- | --- |
| Tree Height | 27.5 | 21.0 | 36.6 | 7995 |
| Clock Rate | 2.4 × 10^-3^ | 1.7 × 10^-3^ | 3.2 × 10^-3^ | 8967 |
| Substitution rate =(Clock Rate * 171) | 0.41 | 0.29 | 0.55 | / |
| Kappa | 5.9 | 4.2 | 8.2 | 8419 |
| Ne Badger | 34 | 20 | 51 | 7777 |
| Ne Cattle | 1.2 | 0.27 | 2.7 | 7701 |
| Cattle to Badger migration rate | 0.042 | 7.0 × 10^-6^ | 0.24 | 8736 |
| Badger to cattle migration rate | 2.2 | 0.79 | 4.7 | 8060 |
