# Supplementary figures and images for: A Bayesian evolutionary model towards understanding wildlife contribution to F4-family Mycobacterium bovis transmission in the South-West of France
Source: Vet Res. 2022 Apr 2;53:28. doi: 10.1186/s13567-022-01044-x (PMC8976416; doi:10.1186/s13567-022-01044-x)

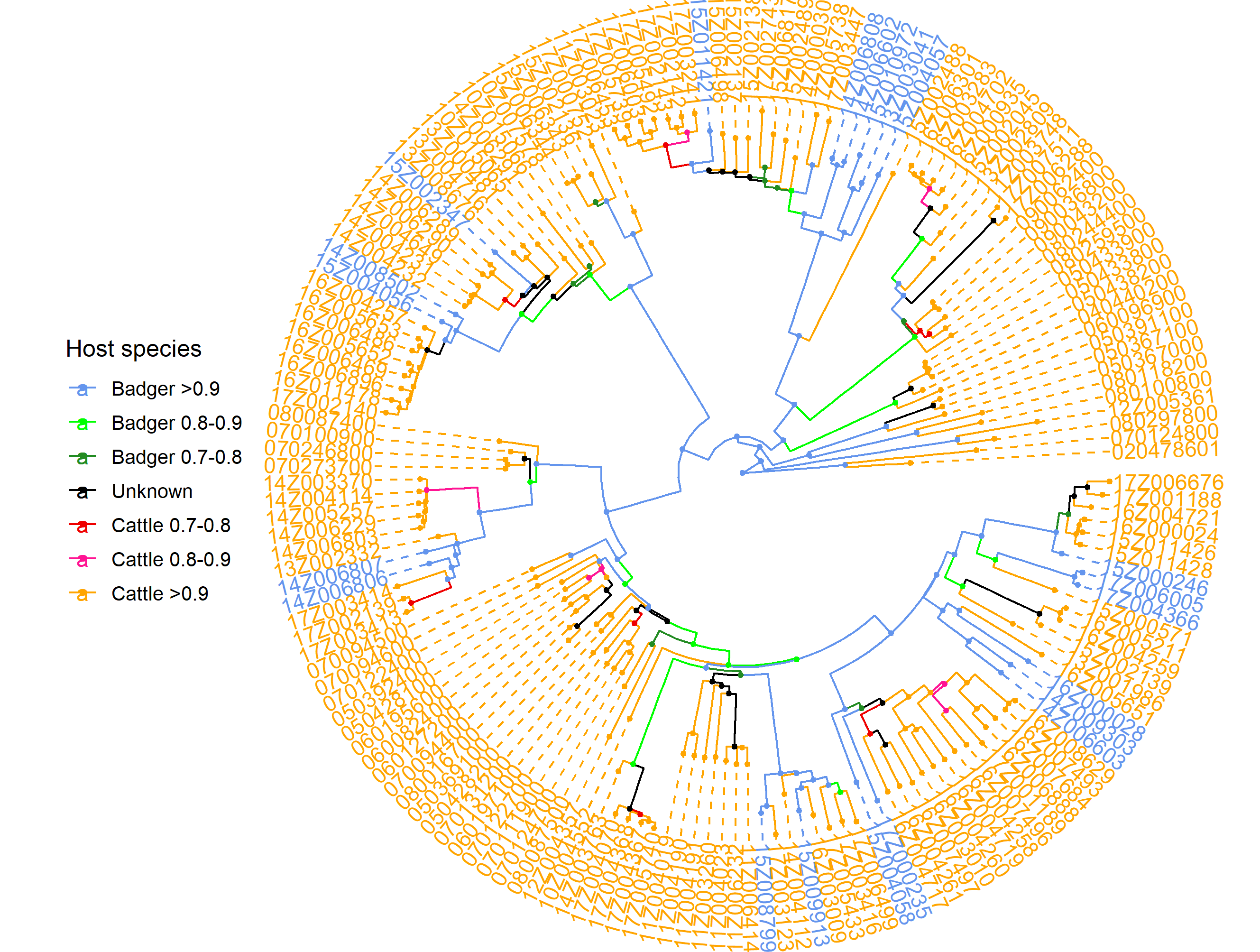

Supplement: Supplementary file 3 — Additional file 3: Maximum Clade Credibility (MCC) tree reconstructed with strain names. Colors represent either host-species, in which the strains were isolated (for tree tips) or the reconstructed host-species (internal nodes). Host-species are considered unknown if the host probability is inferior to 0.70. [file 13567_2022_1044_MOESM3_ESM.tiff]

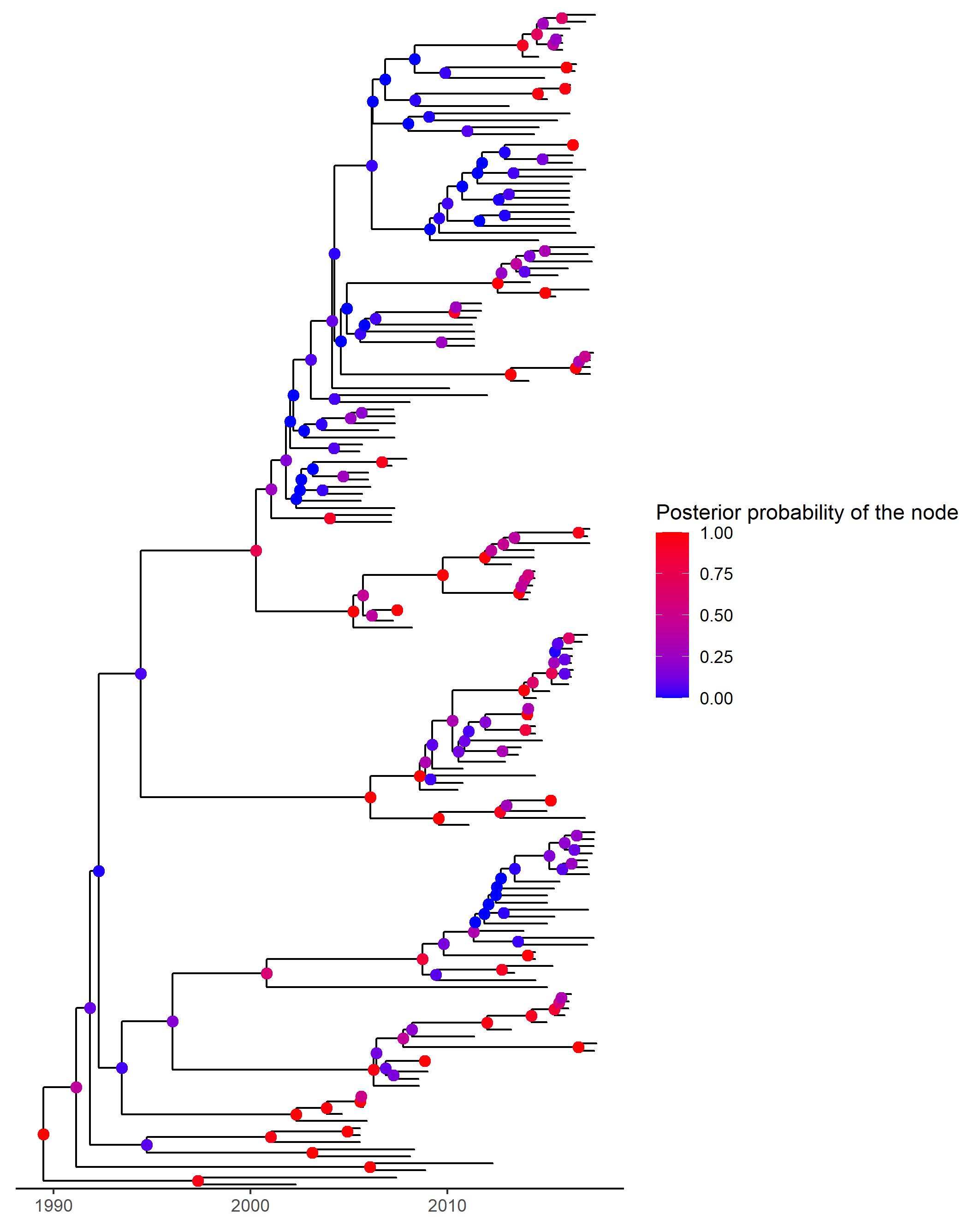

Supplement: Supplementary file 4 — Additional file 4: Maximum Clade Credibility (MCC) tree reconstructed with posterior probabilities. Colors represent the posterior probability of the nodes. [file 13567_2022_1044_MOESM4_ESM.tiff]
