# Supplementary material for: A Bayesian evolutionary model towards understanding wildlife contribution to F4-family Mycobacterium bovis transmission in the South-West of France
Source: Vet Res. 2022 Apr 2;53:28. doi: 10.1186/s13567-022-01044-x (PMC8976416; doi:10.1186/s13567-022-01044-x)
Supplement: Supplementary file 5 — Additional file 5: Number of inter-species transitions per tree calculated over 1004 sampled trees. Number of transitions are represented according to transition type and various probability thresholds (0.7, 0.8 and 0.9). [file 13567_2022_1044_MOESM5_ESM.docx]

| **Transition type** | **Badger to Badger** | | | **Badger to Cattle** | | | **Cattle to Badger** | | | **Cattle to Cattle** | | |
| --- | --- | --- | --- | --- | --- | --- | --- | --- | --- | --- | --- | --- |
| Probability threshold | 0.7 | 0.8 | 0.9 | 0.7 | 0.8 | 0.9 | 0.7 | 0.8 | 0.9 | 0.7 | 0.8 | 0.9 |
| Minimum | 11 | 9 | 2 | 2 | 1 | 0 | 0 | 0 | 0 | 68 | 66 | 64 |
| 1^st^ quartile | 54 | 38 | 14 | 38 | 26 | 10 | 0 | 0 | 0 | 86 | 81 | 78 |
| Median | 118 | 115 | 109 | 77 | 72 | 64 | 1 | 1 | 0 | 120 | 116 | 112 |
| 3^rd^ quartile | 140 | 139 | 137 | 97 | 95 | 91 | 8 | 5 | 3 | 176 | 169 | 158 |
| Maximum | 150 | 149 | 148 | 106 | 104 | 103 | 12 | 9 | 7 | 273 | 256 | 241 |
